# A Novel Adaptive Robust Cubature Kalman Filter for Maneuvering Target Tracking with Model Uncertainty and Abnormal Measurement Noises

**DOI:** 10.3390/s23156966

**Published:** 2023-08-05

**Authors:** Xiangzhou Ye, Jian Wang, Dongjie Wu, Yong Zhang, Bing Li

**Affiliations:** 1 Key Laboratory of Infrared System Detection and Imaging Technology, Chinese Academy of Sciences, Shanghai 200083, China; yexiangzhou@mail.sitp.ac.cn (X.Y.); wangjian@mail.sitp.ac.cn (J.W.); wudongjie@mail.sitp.ac.cn (D.W.); zhangyong@mail.sitp.ac.cn (Y.Z.); 2 Shanghai Institute of Technical Physics, Chinese Academy of Sciences, Shanghai 200083, China; 3 University of Chinese Academy of Sciences, Beijing 100049, China

**Keywords:** target tracking, H-infinity cubature Kalman filter, adaptive fading factor, Sage–Husa

## Abstract

The features of measurement and process noise are directly related to the optimal performance of the cubature Kalman filter. The maneuvering target model’s high level of uncertainty and non-Gaussian mean noise are typical issues that the radar tracking system must deal with, making it impossible to obtain the appropriate estimation. How to strike a compromise between high robustness and estimation accuracy while designing filters has always been challenging. The H-infinity filter is a widely used robust algorithm. Based on the H-infinity cubature Kalman filter (HCKF), a novel adaptive robust cubature Kalman filter (ARCKF) is suggested in this paper. There are two adaptable components in the algorithm. First, an adaptive fading factor addresses the model uncertainty issue brought on by the target’s maneuvering turn. Second, an improved Sage–Husa estimation based on the Mahalanobis distance (MD) is suggested to estimate the measurement noise covariance matrix adaptively. The new approach significantly increases the robustness and estimation precision of the HCKF. According to the simulation results, the suggested algorithm is more effective than the conventional HCKF at handling system model errors and abnormal observations.

## 1. Introduction

Target tracking is estimating motion parameters such as the tracked target’s position and velocity in real time through measurement data [1]. It is a prerequisite for following tasks like target recognition and data fusion. Radar tracking is the primary method of target tracking. There are numerous methods, including infrared tracking, laser tracking, sonar tracking, and other types of tracking, due to the development of associated technologies, the diversity of the types of tracked objects, and the complexity of motion [2]. Target tracking plays an essential role in many fields, such as vehicle monitoring [3], space attitude perception [4], and missile guidance [5]. Many literature studies focus on efficiently tracking high-speed maneuvering targets with fast flight speeds and complex trajectories [6,7,8,9,10].

The Kalman filter, an ideal recursive estimator created for linear Gaussian systems, is the approach for target tracking that is most frequently used [11]. However, the system is frequently nonlinear in practical engineering applications, particularly the measurement equation. Scholars have proposed the extended Kalman filter (EKF), based on Taylor series expansion, and the unscented Kalman filter (UKF), based on sampling distance estimation, to suit the needs of practical nonlinear filtering better [12]. However, the EKF has disadvantages such as poor stability, imprecision, and sluggish target maneuver reaction. When faced with high-dimensional system states, the UKF is vulnerable to dimensional disasters, which cause a sharp reduction in filtering performance and cause it to fall behind the target [13]. Regarding numerical accuracy and operational stability, the cubature Kalman filter (CKF) [14] suggested by Arasaratnam is preferable to the algorithm mentioned above. The sigma point sampling approach and weight distribution of the UKF are optimized with the CKF using spherical integral and radial integral criteria, which resolves the dimension disaster issue. The CKF is a case of UKF when the free parameter equals zero. It offers a rigorous theoretical foundation for zero degrees of freedom in high-dimensional state estimation. However, maneuvering targets usually do not maintain only one motion mode, and the trajectories of maneuvering targets within the military domain exhibit a heightened level of complexity, compounded by an unpredictable operational environment, which often gives rise to indeterminate measurement noise. Therefore, the CKF, like other nonlinear filters, will suffer a severe decline in tracking accuracy when tracking targets under such conditions.

An H-infinity filter not based on the assumption of signal spectral characteristics is proposed in [15] to increase the robustness of navigation and tracking systems. The H-infinity filter is designed to minimize the impact of the worst disturbance on the estimation error by incorporating the H-infinity norm into the filter design. In [16,17], the H-infinity filter, which can be applied only to linear systems, is applied to nonlinear systems, maintaining the advantages of the CKF and H-infinity filter. However, various circumstances, including model uncertainty and external interference, will prevent information from fusing, lowering the H-infinity filter’s estimation accuracy.

Academics have presented some adaptive techniques to further enhance the filtering algorithm’s estimation performance. Zhou proposed a strong tracking filter (STF) that can maintain the residual sequence orthogonal to solve the system model uncertainty problem [18]. The STF method can be more robust when the system parameters or state change. The authors of [19,20] proposed an improved STF method based on a point estimation nonlinear Kalman filter and confirmed its effectiveness in navigation tracking systems. The Sage–Husa adaptive Kalman filter (SHAKF), based on maximum a posteriori estimation, is a straightforward and helpful solution for the uncertain noise in the measurement process [21]. The SHAKF algorithm can estimate the statistical characteristics of noise in real time and reduce the filtering divergence. However, ensuring that SHAKF will calculate the noise covariance matrix in a positive, definite manner can be challenging, which could result in filtering divergence. Performance will also decrease if measurement noise is mixed with non-Gaussian noise. The Mahalanobis distance (MD) is an index for anomaly statistical detection [22]. The robust estimate approach can be used for robust filtering when the observation value is abnormal, which can successfully lessen the impact of abnormal model deviation and abnormal measurement [23].

The research presented in this paper develops an adaptive fading factor and noise covariance estimation method with a robust strategy based on the traditional HCKF to solve the issues mentioned earlier. A low-cost STF calculation method is adopted to solve the problem of insufficient tracking accuracy caused by model errors. The MD-improved Sage–Husa estimation method is used to estimate abnormal or non-Gaussian measurement noise, further improving the robustness and tracking accuracy of the HCKF. The major contributions of this paper can be summarized as follows:

1. A low-cost strong tracking fading factor is proposed. This method avoids the calculation of the Jacobian matrix and additional sampling operations, significantly reducing the algorithm’s time complexity. At the same time, it also solves the problem of STF failure caused by the large difference in the order of magnitude of each dimension of the measurement system.
2. An MD-based method is proposed to correct the noise covariance of the Sage–Husa estimate. Sage–Husa estimation decreases measuring system error by estimating measurement noise in real time, but this method can easily lead to non-positive definite matrices and filter divergence. The MD approach is used in this study to construct the expansion factor, which is used to correct the measurement noise updated by the Sage–Husa estimate, limit the interference of outliers on the filter results, and improve the algorithm’s robustness.
3. For the composite problems of model mismatch and measurement noise anomaly that may occur in maneuvering target tracking, a new robust adaptive cubature Kalman filter is proposed. The new method, which is based on the HCKF algorithm, introduces the two new, enhanced methods mentioned above, and suppresses the influence of system mutation and non-Gaussian noise. The simulation results show that the proposed method achieves a similar impact as the interacting-multiple model CKF (IMMCKF) algorithm in the face of a sudden change in maneuvering target motion. It also exhibits improved robustness and tracking accuracy in the presence of anomalous measurement noise compared with the CKF, HCKF, and Sage–Husa CKF.

The rest of this article is organized as follows. Section 2 describes the system model and the problems to be solved. Section 3 gives the principle and process steps of the traditional HCKF algorithm. Section 4 introduces an improved adaptive robust cubature Kalman filter for high-speed maneuvering target tracking. In Section 5, the proposed algorithm’s effectiveness is verified with various simulation experiments. Finally, the conclusion is followed in Section 6.

## 2. Problem Formulation

Consider the following nonlinear discrete system:

(1) $$X_{k}=f\left(X_{k-1}\right)+v_{k-1}$$

(2) $$Z_{k}=h\left(X_{k}\right)+w_{k}$$

where $X_{k}\in R^{n}$ is the system state quantity at time *k*; $Z_{k}\in R^{m}$ is the system measurement at time *k*; $f\left(\cdot \right)$ and $h\left(\cdot \right)$ are the nonlinear functions of the system state equation and the measurement equation, respectively; $v_{k-1}$ and $w_{k}$ represent the process noise and measurement noise of the system; the mean is zero; and the variances are $Q_{k-1}$ and $R_{k}$, respectively.

According to the classic CKF, the system must be accurately modeled, and the process and measurement noise must be Gaussian white noise with well-known statistical properties. The process state equation will unavoidably vary in the radar monitoring system due to the maneuvering target’s easily modifiable trajectory. The measurement noise is typically non-standard Gaussian noise due to external environment disturbance, and its statistical features cannot be recognized in real time. The traditional filter algorithm will not be able to estimate the state accurately in this situation, and divergence issues may even arise.

The problems solved in this paper are described as follows:

1. The high-speed maneuvering target has uncertain reentry motion, which is the main reason for the significant decrease in tracking accuracy. An improved algorithm is needed to solve the model error caused by maneuvering motion.
2. The statistical characteristics of non-Gaussian noise cannot be accurately estimated in real time, and it is necessary to improve the system’s resistance to unknown noise as much as possible.
3. Due to parameter settings, the proposed improvement approaches would interact with one another during implementation, necessitating specific designs to avoid system interference.

## 3. The H-Infinity Cubature Kalman Filter

The H-infinity filter is based on game theory, which aims to minimize the estimation error for all disturbances with bounded energy. It is a particular form of Kalman filter [15]. For the nonlinear discrete system proposed in the previous section, the H-infinity filter design idea is that when $P_{k}$, $Q_{k-1}$, and $R_{k}$ reach the upper limit, there is a specific cost function to minimize [24]:

(3) $$J_{\infty }=\frac{\sum_{k=1}^{N}\left.|X_{k}-{\hat{x}}_{k}\right.{|}_{P_{k}^{-1}}^{2}}{\left.|X_{0}-{\hat{x}}_{0}\right.{|}_{P_{0}^{-1}}^{2}+\sum_{k=1}^{N}\left(\left.|v_{k}\right.{|}_{Q_{k}^{-1}}^{2}+\left.|w_{k}\right.{|}_{R_{k}^{-1}}^{2}\right)}$$

where $P_{k}$ represents the posterior covariance matrix and $P_{0}$ and $X_{0}$ are the initial covariance matrix and the system’s initial state, respectively. The standard symbol operations used in the formula are as follows: $\left.|x\right.{|}_{A}^{2}=x^{T}Ax$.

The purpose of the H-infinity filter is to estimate ${\hat{x}}_{k}$ minimized $J_{\infty }$. In general, the analytical solution of the optimal H-infinity filter problem is challenging to obtain, so the suboptimal solution is generally sought [25]. In the worst case, the boundary of $J_{\infty }$ satisfies:

(4) $$\sup\left(J_{\infty }\right)<\gamma^{2}$$

where sup represents the upper bound and γ is a positive scalar parameter that limits the estimation error of the system due to uncertainty. Based on Equation (4), the designer must find ${\hat{x}}_{k}$ such that $\sup\left(J_{\infty }\right)<\gamma^{2}$ holds for any perturbation in $v_{k}$, $w_{k}$, and $X_{0}$.

The cubature Kalman filter is a nonlinear filter based on the third-order spherical-radial cubature rule proposed by Arasaratnam [14]. The CKF regards the estimation of nonlinear equations as the estimation of a probability distribution based on Bayesian estimation, which dramatically simplifies the nonlinear problem and can be well applied to high-dimensional nonlinear system filtering [26]. In order to apply H-infinity filtering to nonlinear systems, a cubature Kalman filter (HCKF) based on H-infinity filtering is proposed [16,27]. The algorithm steps are as follows:

Prediction Update:

(1) The system’s initial predictive value and covariance matrix are set to ${\hat{x}}_{0}$ and $P_{0}$. Calculate 2*n* cubature points $X_{i,cub}$ from ${\hat{x}}_{k-1}$ and $P_{k-1}$:

(5) $$X_{i,cub}=S_{k-1}\xi_{i}+{\hat{x}}_{k-1}$$

where $S_{k-1}$ is obtained with Cholesky decomposition of $P_{k-1}$. The cubature points $\xi_{i}=\sqrt{n}{\left[I_{n},-I_{n}\right]}_{i}$ and $I_{n}$ represent the *n*-dimensional unit matrix.

The obtained cubature points $X_{i,cub}$ are propagated through the nonlinear state function:

(6) $$X_{i,k|k-1}^{*}=f\left(X_{i,cub}\right)$$

(2) The prior prediction state value ${\hat{x}}_{k|k-1}$ and the prediction covariance matrix $P_{k|k-1}$ are calculated.

(7) $${\hat{x}}_{k|k-1}=\omega \sum_{i=1}^{2n}X_{i,k|k-1}^{*}$$

(8) $$P_{k|k-1}=\omega \sum_{i=1}^{2n}X_{i,k|k-1}^{*}{\left(X_{i,k|k-1}^{*}\right)}^{T}-{\hat{x}}_{k|k-1}{\hat{x}}_{k|k-1}^{T}+Q_{k-1}$$

where ω=1/2n is the cubature points’ weight.

Measurement Update:

(3) The cubature points are calculated again according to the prior prediction ${\hat{x}}_{k|k-1}$ and the prediction covariance matrix $P_{k|k-1}$ obtained in the previous step, and ${\hat{z}}_{k|k-1}$ is obtained by nonlinear measurement function propagation.

(9) $$P_{k|k-1}=S_{k|k-1}S_{k|k-1}^{T}\;$$

(10) $$X_{i,cub}^{*}=S_{k|k-1}\xi_{i}+{\hat{x}}_{k|k-1}$$

(11) $$Z_{i,k|k-1}^{*}=h\left(X_{i,cub}^{*}\right)$$

(12) $${\hat{z}}_{k|k-1}=\omega \sum_{i=1}^{2n}Z_{i,k|k-1}^{*}$$

(4) The autocorrelation covariance matrix $P_{zz}$ and the cross-correlation covariance matrix $P_{xz}$ are as follows:

(13) $$P_{zz}=\omega \sum_{i=1}^{2n}Z_{i,k|k-1}^{*}{\left(Z_{i,k|k-1}^{*}\right)}^{T}-{\hat{z}}_{k|k-1}{\hat{z}}_{k|k-1}^{T}+R_{k}=P_{ee}+R_{k}$$

(14) $$P_{xz}=\omega \sum_{i=1}^{2n}X_{i,k|k-1}^{*}{\left(Z_{i,k|k-1}^{*}\right)}^{T}-{\hat{x}}_{k|k-1}{\hat{z}}_{k|k-1}^{T}$$

(5) Calculate the Kalman gain $K_{k}$ according to $P_{zz}$ and $P_{xz}$:

(15) $$K_{k}=P_{xz}P_{zz}^{-1}$$

(6) Finally, the update state ${\hat{x}}_{k}$ and update covariance matrix $P_{k}$ of the system are calculated:

(16) $${\hat{x}}_{k}={\hat{x}}_{k|k-1}+K_{k}\left(Z_{k}-{\hat{z}}_{k|k-1}\right)$$

(17) $$P_{k}=P_{k|k-1}-\left[P_{xz},P_{k|k-1}\right]R_{e,k}^{-1}{\left[P_{xz},P_{k|k-1}\right]}^{T}$$

where

$$R_{e,k}=\left[\begin{array}{cc} P_{zz}+R_{k} & P_{xz}^{T}\\ P_{xz} & P_{k|k-1}-\gamma^{2}I \end{array}\right]$$

The threshold γ determines the robustness of the filter. When it approaches infinity, the HCKF degenerates into the ordinary CKF. In other words, the Kalman filter is a particular case of an H-infinity filter when its performance boundary is infinite. The adversary in Kalman filtering is considered irrelevant from the game theory standpoint. The probability distribution function of the noise is known when designing a Kalman filter, and it may be used to create a statistically optimal state estimation. At the same time, the adversary does not alter this probability distribution function to degrade the performance of the estimated state. Therefore, while the Kalman filter minimizes the variance of the estimation error, it does not guarantee the estimation error in the worst case; that is, it does not ensure the boundary of the cost function in Equation (3). The H-infinity filter equation has a more unambiguous interpretation than the Kalman filter equation. It is a filter that evaluates the worst-case scenario. The opponent can maximize the estimating error by just using the infinite $X_{0}$, $w_{k}$, and $v_{k}$, which makes the game unfair. Thus, when defining $J_{\infty }$, $X_{0}-{\hat{x}}_{0}$, $w_{k}$, and $v_{k}$ are in the denominator. Even if using infinite $X_{0}$, $w_{k}$, and $v_{k}$ increases the estimation error, the $J_{\infty }$ may not increase because the denominator also increases. Because the form of $J_{\infty }$ prohibits the adversary from utilizing cruel means to maximize the estimation error, the architecture of the H-infinity filter is robust.

## 4. Adaptive Robust H-Infinity Cubature Kalman Filter

The HCKF requires that all parameters and noise statistics of the system model must be known, which is almost impossible in actual maneuvering target tracking. The trajectory of a maneuvering target usually cannot be represented by a single kinematic model, and the measurement system’s noise is usually heavy tailed. The above problems will lead to a decrease in the estimation accuracy of the filter.

In order to compensate for the modeling error and improve the ability of the filter to deal with abnormal measurements, we introduce the STF and the Sage–Husa noise estimator method based on Section 3 and propose a new adaptive robust cubature Kalman filter (ARCKF). The STF corrects the prediction error covariance matrix by introducing an adaptive fading factor to compensate for the modeling error and maintain the excellent performance of the filter [28]. The Sage–Husa method is based on maximum a posteriori estimation, which has a simple structure and good real-time performance. It is often used to estimate the statistical characteristics of noise [29].

### 4.1. Fading Factor Based on Strong Tracking Filter

The strong tracking filter [18] is an algorithm based on EKF. This algorithm is based on the orthogonality principle of output residual sequence. It introduces the adaptive fading factor into the state prediction covariance matrix, which solves the problem that the EKF cannot converge when the model error is significant. The strong tracking filter mainly modifies the prediction covariance matrix $P_{k|k-1}$ by satisfying the following two equality conditions:

(18) $$E\left[\left(X_{k}-{\hat{x}}_{k}\right){\left(X_{k}-{\hat{x}}_{k}\right)}^{T}\right]=min$$

(19) $$E\left(\varepsilon_{k+j}\varepsilon_{k}^{T}\right)=0,\left(k=0,1,\ldots ;j=1,2,\ldots \right)$$

where ${\hat{x}}_{k}$ is the state estimation at time *k*, $\varepsilon_{k}=Z_{k}-{\hat{z}}_{k|k-1}$ is the output residual at time *k*, and ${\hat{z}}_{k|k-1}$ is the observation prediction at time *k*. Equations (18) and (19) are sufficient conditions for STF. These two conditions are called the orthogonality principle. The filtering performance index under the minimum variance estimation criterion is given by Equation (18), which states that the output of the filter must meet the minimal mean square error; Equation (19) shows that the residual sequence is orthogonal. That is, the residual sequences at different times are not related. When the filter has a model mismatch problem, the estimated value of the system state deviates from the actual state of the system, which is reflected in the residual sequence. By adjusting the Kalman gain in real time to meet Equation (19), it can ensure that the residual sequence always has Gaussian white noise characteristics and improve the tracking ability of the system; when the system model is accurate, Equation (19) is satisfied, and the filter is equivalent to the original filter.

Equation (19) is the core of the orthogonality principle. Equation (18) represents the design standard the original filter must meet. When the original filter is combined with the criteria of Equation (19), it exhibits the features of the STF. The STF modifies the prediction covariance matrix to update the Kalman gain using the adaptive fading factor$\;\lambda_{k}$. The following is the computation method [28]:

(20) $$\lambda_{k}=\max\left(\lambda_{0},1\right)$$

(21) $$\lambda_{0}=\frac{tr\left[N_{k}\right]}{tr\left[M_{k}\right]}$$

(22) $$N_{k}=V_{k}-C_{k}Q_{k-1}C_{k}^{T}-R_{k}$$

(23) $$M_{k}=C_{k}F_{k}P_{k-1}F_{k}^{T}C_{k}^{T}$$

(24) $$V_{k}=\left\{\begin{array}{cc} \varepsilon_{1}\varepsilon_{1}^{T} & \left(k=1\right)\\ \frac{\rho V_{k}+\varepsilon_{k}\varepsilon_{k}^{T}}{1+\rho } & \left(k>1\right) \end{array}\right.$$

where $F_{k}$ and $C_{k}$ are the Jacobian matrices after the first-order Taylor expansion of the state function and the measurement function, respectively; $\rho \left(0<\rho \leq 1\right)$ represents the forgetting factor, usually 0.95. The computational complexity of Jacobian matrix calculation is high, and this method, using first-order Taylor expansion approximation, only applies to weakly nonlinear systems, which significantly limits the application scenarios of the STF. Moreover, the fading factor calculates the ratio of the eigenvalues of each dimension element of the measurement residual matrix to the theoretical output value of the filter. This calculation method brings problems to the measurement system with significant differences in the order of magnitude of each dimension. For example, the angle residual in the radar tracking system is much smaller than the distance residual. When the maneuvering target is mainly maneuvering at the angle, the small change in the angle residual may lead to the failure of the fading factor. Therefore, it is necessary to improve the calculation of the fading factor to better cope with the above problems.

Applying statistical linearization to the nonlinear observation equation [30], we can obtain

(25) $$z_{k}=H_{k}\left(x_{k}-{\hat{x}}_{k|k-1}\right)+{\hat{z}}_{k|k-1}+v_{k}$$

where $H_{k}=P_{xz}^{T}P_{k|k-1}^{-1}$ is the statistical regression equation.

According to the theorem in [18], the sufficient condition for the orthogonality of the output residual sequence is:

(26) $$P_{k|k-1}H_{k}^{T}-K_{k}V_{k}=0$$

Using the definition of the cross-correlation covariance matrix $P_{xz}$, we can obtain:

(27) $$\begin{array}{cc} P_{xz} & =E\left[\left(x_{k}-{\hat{x}}_{k|k-1}\right){\left(z_{k}-{\hat{z}}_{k|k-1}\right)}^{T}\right]\\ & =E\left[\left(x_{k}-{\hat{x}}_{k|k-1}\right){\left(H_{k}\left(x_{k}-{\hat{x}}_{k|k-1}\right)+v_{k}\right)}^{T}\right]\\ & =E\left[\left(x_{k}-{\hat{x}}_{k|k-1}\right){\left(x_{k}-{\hat{x}}_{k|k-1}\right)}^{T}H_{k}^{T}\right]=P_{k|k-1}H_{k}^{T} \end{array}$$

Equation (26) is rewritten as:

(28) $$P_{xz}-K_{k}V_{k}=P_{xz}-P_{xz}P_{zz}^{-1}V_{k}=0$$

(29) $$P_{zz}-V_{k}=0$$

It is known that the autocorrelation covariance matrix has the form

(30) $$\begin{array}{cc} P_{zz} & =E\left[\left(z_{k}-{\hat{z}}_{k|k-1}\right){\left(z_{k}-{\hat{z}}_{k|k-1}\right)}^{T}\right]\\ & =E\left[\left(H_{k}\left(x_{k}-{\hat{x}}_{k|k-1}\right)+v_{k}\right){\left(H_{k}\left(x_{k}-{\hat{x}}_{k|k-1}\right)+v_{k}\right)}^{T}\right]\\ & =H_{k}P_{k|k-1}H_{k}^{T}+R_{k} \end{array}$$

The fading factor $\lambda_{k}$ is introduced to correct $P_{k|k-1}$ to ensure that the performance index of Equation (29) is satisfied. Therefore, Equation (29) is further rewritten as:

(31) $$\lambda_{k}\left(P_{zz}-R_{k}\right)+R_{k}=V_{k}$$

Redefining $M_{k}$ and $N_{k}$ in accordance with Equation (31),

(32) $$N_{k}=V_{k}-R_{k}$$

(33) $$M_{k}=P_{zz}-R_{k}=P_{ee}$$

where $P_{ee}$ was defined in Equation (13).

Therefore, rather than using Equations (22) and (23), we can determine the fading factor $\lambda_{k}$ using Equations (32) and (33). The original method of calculating the fading factor is to compare the trace of the measurement residual to the theoretical output value (see Equation (21)). Algorithm failure may happen when measurement systems have significant variances in the order of magnitude of each dimension. In order to ensure that the fading factor has the same sensitivity to different measurement dimensions, the maximum ratio of different measurement dimensions is considered the fading factor. Then Equation (21) is changed to

(34) $$\lambda_{0}=\max\left\{\frac{{\left[diag\left(N_{k}\right)\right]}_{i}}{{\left[diag\left(M_{k}\right)\right]}_{i}}\right\},i=1,2,\ldots ,m$$

### 4.2. Robust Adaptive Strategy for Measurement Noise Estimator

The adaptive method for estimating the $R_{k}$ matrix is a scaling method based on residual covariance. It is calculated based on the difference between the actual measured value obtained at time *k* and its estimated value. The recursive formula of the measurement noise is expressed as [31]:

(35) $$R_{k}=\left(1-d_{k-1}\right)R_{k-1}+d_{k-1}\left(\varepsilon_{k}\varepsilon_{k}^{T}-P_{ee}\right)$$

where

(36) $$d_{k}=\frac{1-b}{1-b^{k+1}},0<b<1$$

$d_{k}$ is a weighted coefficient to measure the influence of noise on the observed value, and *b* represents the forgetting factor. The larger the value is, the stronger the influence of the last measurement is. However, if the value of *b* is too small, the predicted noise will oscillate. The forgetting factor *b* in this method is often set between 0.95 and 0.99 based on numerical simulation attempts or experience (the frequency of changes in noise statistics). The noise estimation in Equation (36) still needs to satisfy the Gaussian distribution, and its resistance to abnormal noise needs to be increased. In order to further improve the robust performance of the HCKF, the square of the Mahalanobis distance from $Z_{k}$ to ${\hat{z}}_{k|k-1}$ is considered the criterion [32]. The formula is as follows:

(37) $$MD_{k}^{2}={\left(\sqrt{{\left(Z_{k}-{\hat{z}}_{k|k-1}\right)}^{T}{\left(P_{zz}\right)}^{-1}\left(Z_{k}-{\hat{z}}_{k|k-1}\right)}\right)}^{2}=\varepsilon_{k}^{T}{\left(P_{ee}+R_{k}\right)}^{-1}\varepsilon_{k}$$

If the actual criterion index satisfies $MD_{k}^{2}>\chi_{n,\alpha }^{2}$, then the observed value $Z_{k}$ is marked as an outlier, and the expansion factor μ also amplifies the covariance of the observed noise; that is,

(38) $${\tilde{R}}_{k}=\mu R_{k}$$

at this time should satisfy

(39) $${MD}_{k}^{2}={\varepsilon_{k}^{T}\left(P_{ee}+{\tilde{R}}_{k}\right)}^{-1}\varepsilon_{k}=\chi_{n,\alpha }^{2}$$

where $\chi_{n,\alpha }^{2}$ is the statistical detection threshold conforming to the chi-square distribution and $\chi_{n,\alpha }^{2}=\chi^{2}\left(n\right)$. The problem of solving $\mu_{k}$ can be transformed into the following nonlinear equation:

(40) $$f\left(\mu_{k}\right)={\varepsilon_{k}^{T}\left(P_{ee}+{\tilde{R}}_{k}\right)}^{-1}\varepsilon_{k}-\chi_{n,\alpha }^{2}=0$$

Considering the use of a Newton iterative method to solve $\mu_{k}$ in Equation (40), the recursive relationship can be expressed as follows:

(41) $$\mu_{k}\left(i+1\right)=\mu_{k}\left(i\right)+\frac{MD_{k}^{2}\left(i\right)-\chi_{n,\alpha }^{2}}{{\varepsilon_{k}^{T}\left({\tilde{P}}_{zz}\left(i\right)\right)}^{-1}R_{k}{\left({\tilde{P}}_{zz}\left(i\right)\right)}^{-1}\varepsilon_{k}}$$

where *i* represents the number of iterations, ${\tilde{P}}_{zz}\left(i\right)=P_{ee}+\mu_{k}\left(i\right)R_{k}$, we set the initial value of the iteration to $\mu_{k}\left(0\right)=1$, and the iteration ends only when the decision condition $\mu_{k}\left(i\right)\leq \chi_{n,\alpha }^{2}$ is satisfied. It can be considered that α is set to 0.99, which means that the filter’s efficiency is 99%. According to the look-up table method, the chi-square distribution $\chi_{2,0.99}^{2}$ of 2 degrees of freedom can be determined to be 9.21.

It should be noted that there are many reasons for the abnormal state prediction value. The system’s modeling error and abnormal measurement noise will affect the final state prediction value. The wrong use of the correction matrix ${\tilde{R}}_{k}$ is likely to lead to misjudgment, so that the STF algorithm and the robust estimation method of measurement noise work simultaneously. However, it is easy to lead to the divergence of the filter. Therefore, the calculation of the adaptive fading factor in Equation (34) uses the original $R_{k}\;$instead of the modified ${\tilde{R}}_{k}$ to prevent the ${\tilde{R}}_{k}$ based on the Mahalanobis distance correction from affecting the calculation in the presence of kinematic model errors.

**Remark** **1.** *Adding a fading factor and adjusting measurement noise are performed using a scaling coefficient and positive definite matrix operation. Therefore, both the new approach and the conventional HCKF may guarantee the positive definiteness of the error covariance matrix. Positive definiteness may be lost in practice due to errors brought about by arithmetic operations carried out on a digital computer with a finite word length. The number-sensitive operations that are most likely to destroy the positive definiteness of the covariance include matrix square-rooting [see (5) and (9)] and matrix inversion [see (15) and (17)]. When working with high-precision measurement systems, numerically ill-conditioned is another possibility that could lead to a non-positive definite covariance matrix. The most widely utilized methods to lessen the negative consequences that could cause unstable or even divergent behavior include swapping out the CKF algorithm for SCKF and substituting Cholesky decomposition with SVD decomposition. This paper does not examine this section of the content because it is outside the purview of the work.*

**Figure 1:**
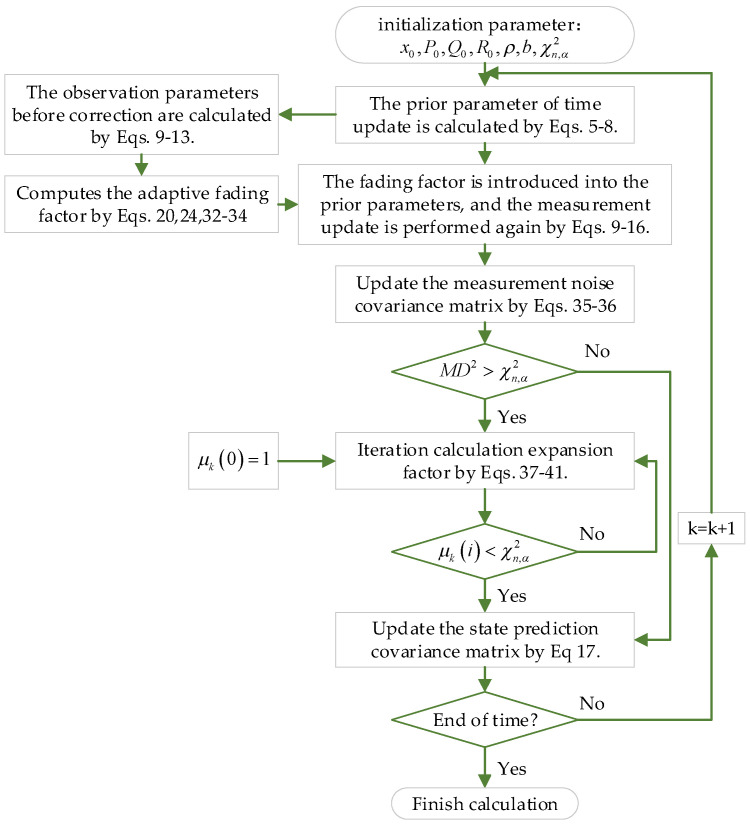
The specific steps of the ARCKF algorithm.

In summary, we give the adaptive robust correction strategy of the HCKF, and the specific process steps of the algorithm are shown in Figure 1.

## 5. Numerical Examples and Analysis

In order to verify the robustness and accuracy of the ARCKF algorithm proposed in this paper, three possible situations in maneuvering target tracking are simulated and compared with the estimation results of the CKF [14], HCKF [27], and Sage–Husa cubature Kalman filter (SHCKF) [29]. It should be noted that in order to maintain robustness, the conventional SHCKF method cannot concurrently perceive process noise and measurement noise. Therefore, we consider improving the SHCKF with the method in Reference [8]. Although this approach trades some noise perception accuracy, it can guarantee that the algorithm will maintain robustness and perform comparative experiments more effectively. In the experiment, 100 Monte Carlo simulations were run to reflect the tracking capability of these algorithms as accurately as possible.

In the simulation experiment, the radar position is set as the origin of the rectangular coordinate system, and the measurement information of the radar is distance and azimuth; then, the corresponding measurement equation is

(42) $$Z_{k}=\left[\begin{array}{c} r_{k}\\ \theta_{k} \end{array}\right]=\left[\begin{array}{c} \sqrt{x^{2}+y^{2}}\\ \arctan\frac{y}{x} \end{array}\right]+w_{k}$$

The state vector of the system is $X={\left[x,v_{x},y,v_{y},\omega \right]}^{T}$, x,y is the position of the maneuvering target in x direction and y direction, $v_{x},v_{y}$ is the velocity of the maneuvering target in x direction and y direction, and ω is the turning rate. In most cases, the maneuvering target always maintains uniform motion during the cruise phase, so the system model considering the filtering algorithm is set to the uniform motion model:

(43) $$F=\left[\begin{array}{ccccc} 1 & T & 0 & 0 & 0\\ 0 & 1 & 0 & 0 & 0\\ 0 & 0 & 1 & T & 0\\ 0 & 0 & 0 & 1 & 0\\ 0 & 0 & 0 & 0 & 1 \end{array}\right]\;$$

The relevant parameters of the simulation experiment are given in Table 1. The simulation results select the root-mean-square error (RMSE) as the performance index. Position RMSE and velocity RMSE are defined as:

(44) $$\begin{array}{c} RMSE_{pos}^{k}=\sqrt{\frac{1}{N}\sum_{j=1}^{N}{\left(x_{k}^{j}-{\hat{x}}_{k}^{j}\right)}^{2}+{\left(y_{k}^{j}-{\hat{y}}_{k}^{j}\right)}^{2}}\\ RMSE_{vel}^{k}=\sqrt{\frac{1}{N}\sum_{j=1}^{N}{\left({v_{x}}_{k}^{j}-{{\hat{v}}_{x}}_{k}^{j}\right)}^{2}+{\left({v_{y}}_{k}^{j}-{{\hat{v}}_{y}}_{k}^{j}\right)}^{2}} \end{array}$$

where *N* is the number of Monte Carlo simulations, *j* is the *j*th Monte Carlo simulation, and *k* is the simulation time. The values $\left(x_{k}^{j},y_{k}^{j}\right)$ and $\left({v_{x}}_{k}^{j},{v_{y}}_{k}^{j}\right)$ represent the true position and velocity of the maneuvering target, respectively; $\left({\hat{x}}_{k}^{j},{\hat{y}}_{k}^{j}\right)$ and $\left({{\hat{v}}_{x}}_{k}^{j},{{\hat{v}}_{y}}_{k}^{j}\right)$ represent the estimated position and velocity of the filter, respectively.

For filters, consistency is equally as crucial as accuracy because it can help us gauge the algorithm’s robustness. The average normalized estimation error square (ANEES), the consistency analysis index, can be calculated as follows [7]:

(45) $$\;ANEES_{k}=\frac{1}{nN}\sum_{i=1}^{N}{\tilde{x}}_{k}^{T}P_{k}^{-1}{\tilde{x}}_{k}$$

where ${\tilde{x}}_{k}$ and $P_{k}$ are the state estimation error and covariance matrix at time *k*, respectively. If $ANEES_{k}\in \left[l_{b},u_{b}\right]$, the filter is considered consistent, where $l_{b}$ and $u_{b}$ are the lower and upper bounds of the acceptance interval, respectively. If $ANEES_{k}<l_{b}$, then the filter is regarded as “pessimistic” (lacking confidence) because the posterior error covariance is very high compared with the true value; if $ANEES_{k}>u_{b}$, then the filter is considered “optimistic” (overconfident), and the covariance $P_{k|k}$ is too small. Different probability intervals can change the upper and lower bounds, but the overall acceptability range is close to 1.

### 5.1. The System Model Does Not Match

**Figure 2:**
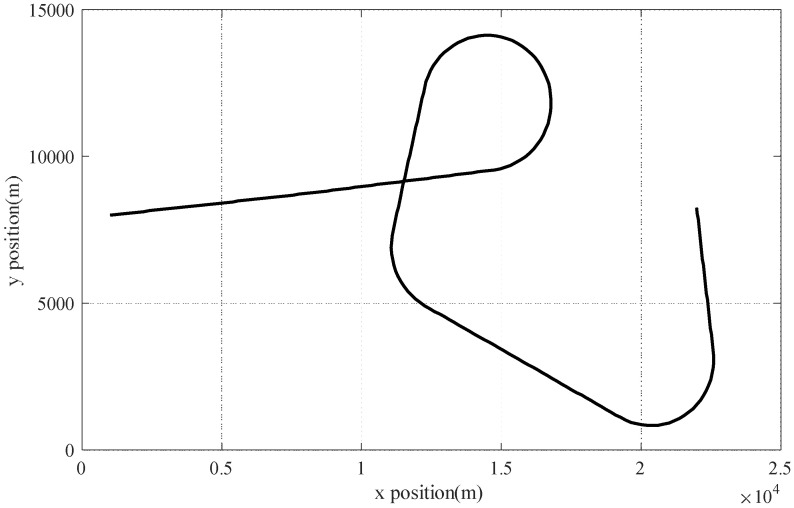
Real trajectory of a high-maneuvering target.

A single kinematic model usually cannot express the motion characteristics of maneuvering targets, which may lead to the loss of targets in the target tracking system when maneuvering orbit changes occur. The interacting-multiple model is a widely used and effective method of dealing with model mismatch issues. As a result, we incorporated IMMCKF as one of the comparison algorithms in this experiment. In this case, the maneuvering target maintains an alternating motion state in 0~250 s, and the periods of uniform motion are 0~70 s, 121~145 s, 161~200 s, and 226~250 s; the periods of maneuver turning are 71~120 s, 146~160 s, and 201~225 s. Among these, the uniform motion section satisfies Equation (43), the maneuvering turning section’s motion equation satisfies Equation (46), and the maneuvering turning rate ω=5°/s. Figure 2 displays the maneuvering target’s trajectory.

(46) $$F_{ct}=\left[\begin{array}{ccccc} 1 & \frac{\sin\omega T}{\omega } & 0 & \frac{\cos\omega T-1}{\omega } & 0\\ 0 & \cos\omega T & 0 & -\sin\omega T & 0\\ 0 & \frac{1-\cos\omega T}{\omega } & 1 & \frac{\sin\omega T}{\omega } & 0\\ 0 & \sin\omega T & 0 & \cos\omega T & 0\\ 0 & 0 & 0 & 0 & 1 \end{array}\right]\;$$

**Figure 3:**
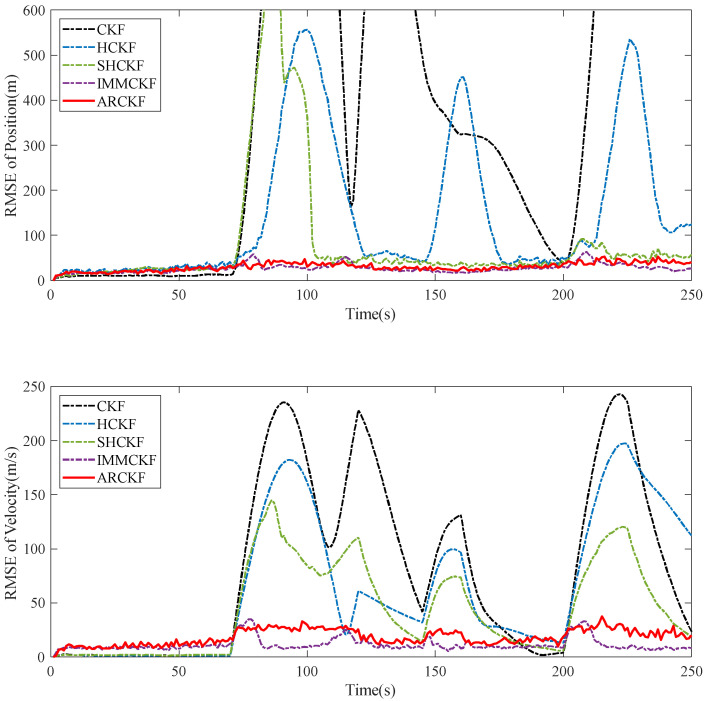
The position and velocity RMSE of each filtering algorithm when the system model does not match.

The RMSE of the position estimate and velocity estimation for various filtering algorithms in Case 5.1 is shown in Figure 3. The filtering algorithm’s model and the initial stage motion are uniform. As a result, these algorithms can produce accurate tracking results. The filtering algorithm’s model and the target’s actual motion diverge significantly when the target maneuvers. The CKF is entirely divergent at this moment and cannot trace the target. The HCKF, based on the CKF, reduces the impact of outliers by a particular function. Although the filter’s robustness is much better than the CKF’s, it still cannot perform effective tracking during the model mismatch stage. The improved SHCKF outperforms the algorithm above at tracking performance. The estimation accuracy of the system noise is nevertheless decreased to prevent algorithm divergence. As a result, when the target changes its movement suddenly, it cannot be rectified in time, and accurate tracking requires some time. By introducing an adaptive fading factor, the ARCKF fully utilizes the useful information in the residual sequence and increases the algorithm’s resistance to uncertainty modeling. The RMSE of the speed estimation only slightly increases when the state changes abruptly, and the RMSE of the position estimation almost wholly ignores the model mismatch. The ARCKF can achieve tracking accuracy similar to the IMMCKF. However, the fundamental model and the Markov transition matrix significantly impact IMMCKF performance. The IMMCKF’s filtering accuracy will be significantly decreased if the trajectory of the maneuvering target does not follow the fundamental model.

### 5.2. Non-Gaussian Measurement Noise

In general, the measurement system is susceptible to abnormal noise pollution. The noise distribution cannot meet the Gaussian distribution and usually presents a heavy-tailed distribution. Contaminated Gaussian distribution noise can be expressed as [22]:

(47) $$w_{k}\sim \left(1-\delta \right)N\left(0,R_{n}\right)+\delta \left(0,R_{p}\right)$$

where $\delta \in \left[0,1\right]$ denotes the proportion of contaminated noise, $R_{n}$ denotes the standard measurement noise error covariance matrix, and $R_{p}$ denotes the contaminated noise covariance matrix, which can be any symmetric distribution. If $R_{p}$ is also a Gaussian distribution with a large standard deviation, the actual noise is also called Gaussian mixed noise, in which the likelihood of abnormal noise grows with an increase in the weight δ of the polluted noise $R_{p}$, and the variance of $R_{p}$ determines the magnitude of the observation deviation produced by the abnormal noise. In this experiment, we assume that $R_{u}=50R_{n}$, and the pollution ratio is 0.2.

**Figure 4:**
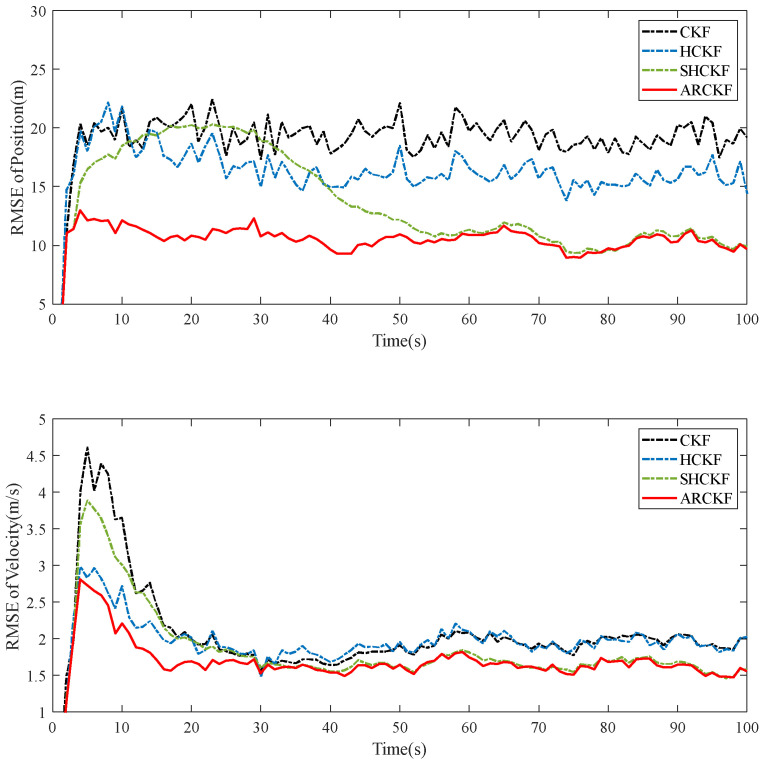
The position and velocity RMSE of each filtering algorithm under non-Gaussian measurement noise.

**Figure 5:**
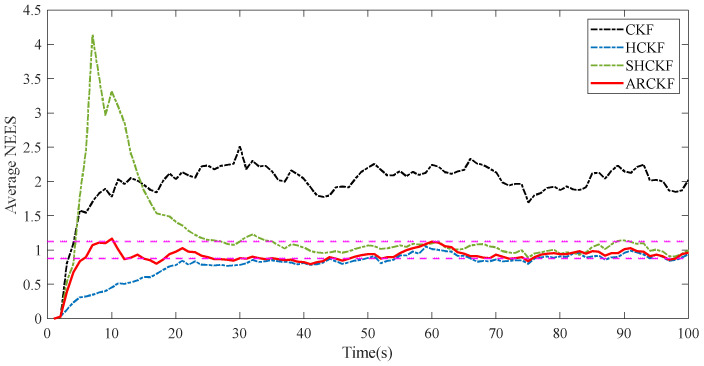
The average NEES of each filtering algorithm under non-Gaussian measurement noise.

Figure 4 shows the RMSE of the position and velocity estimation of different filtering algorithms in Case 5.2. In this case, the HCKF has better filtering accuracy than CKF, converging more quickly during the early filtering stage than CKF; the filtering accuracy of the SHCKF and ARCKF is significantly higher than that of the above two algorithms, but the convergence speed of the SHCKF in the initial stage of filtering is the slowest among the algorithms, which is most obvious in the RMSE of position estimation. The ARCKF retains the advantage that HCKF has the fastest convergence speed in the initial filtering stage. The ARCKF can update the measurement noise covariance in real time, which improves the filtering accuracy of HCKF, and the tracking effect is better than the other three filtering algorithms. Figure 5 shows the estimation consistency of the various filtering methods in Case 5.2. Only the consistency of the CKF is severely impacted by non-Gaussian noise. The other algorithms offer good consistency, but only the ARCKF can do so at the initial filtering stage.

### 5.3. Variable Noise Covariance

When the measurement system tracks the target, sometimes the signal is unstable, which will cause the noise covariance error to change. In this case, we mainly discuss when the noise covariance matrix suddenly increases significantly. The measurement noise is assumed to satisfy the Gaussian distribution, but it will sometimes change:

(48) $$R_{k+1}=\left\{\begin{array}{cc} R_{n} & t<50s\\ {10R}_{n} & t\geq 50s \end{array}\right.$$

where $R_{n}$ represents the standard measurement noise error covariance matrix, and *t* represents the simulation time.

**Figure 6:**
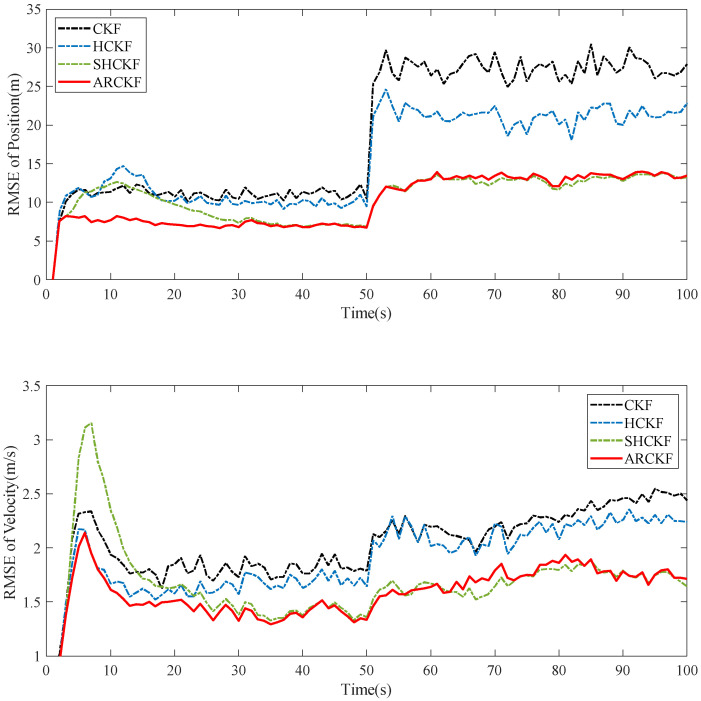
The position and velocity RMSE of each filtering algorithm when the noise covariance is variable.

**Figure 7:**
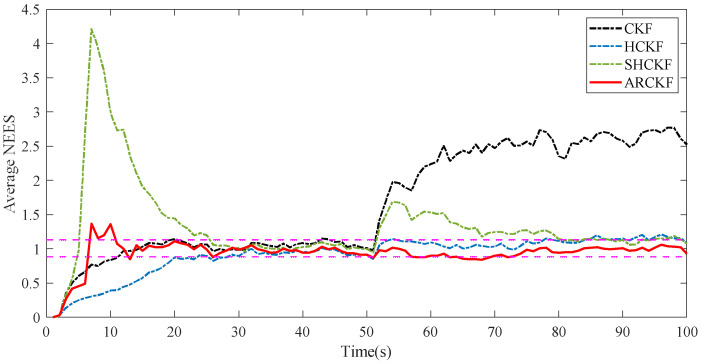
The average NEES of each filtering algorithm when the noise covariance is variable.

Figure 6 shows the RMSE of the position and velocity estimation of the different filtering algorithms in Case 5.3. When the noise covariance matrix becomes larger, the robustness of the four algorithms decreases to a certain extent. However, the SHCKF and ARCKF use the process of adaptively updating the measurement noise covariance. Therefore, these two algorithms have the best resistance to abnormal noise interference after the noise covariance matrix changes. However, when each state changes, the SHCKF always takes a long time to make the algorithm converge. In the face of frequent and complex state changes, the filtering algorithm’s divergence probability will be greatly improved. While using Sage–Husa estimation to update $R_{k}$, the ARCKF introduces the MD method to correct the $R_{k}$ matrix that is easy to diverge, improving the algorithm’s robustness. Figure 7 shows the estimation consistency of the various filtering methods in Case 5.3. The first stage’s actual noise covariance is identical to that of $R_{0}$. These algorithms are relatively consistent, while the CKF and ARCKF can keep consistency more quickly. In the second stage, the measurement noise increases, seriously impairing the consistency of the CKF. Over time, the SHCKF can return to good consistency, but only the HCKF and ARCKF can consistently maintain good consistency.

## 6. Conclusions

An adaptive robust cubature Kalman filter is proposed in this study to address the problems of system model uncertainty and abnormal measurement noise in maneuvering target tracking. Based on the traditional HCKF, the algorithm introduces a simplified adaptive fading factor to solve the problem of system model uncertainty. Sage–Husa estimation is used to update $R_{k}$ in real time to significantly increase the estimation accuracy of the HCKF for the time-varying measurement noise covariance matrix. When an observation value is heavily tailed or anomalous, the MD method includes a scaling factor to lower the observation weight, further enhancing the algorithm’s robustness. The simulation results demonstrate that the proposed ARCKF method has good filtering and estimation performance in tracking moving targets. It can produce results for the model mismatch problem comparable to those of the IMM method, and it also performs better than the CKF, HCKF, and SHCKF algorithms in terms of robustness to non-Gaussian measurement noise.

## Figures and Tables

**Table 1:** Parameters for simulation.

Parameter	Corresponding Value
Number of Monte Carlo simulations	100
Discrete sampling period	*T* = 1 s
Process noise intensities	$q_{1}=0.1\;m^{2}s^{-3},q_{2}=1.75\times 10^{-4\;}s^{-3}$
Initial process noise covariance matrix	$Q_{k-1}=diag\left(\left[q_{1}M_{1},q_{1}M_{1},q_{2}T\right]\right),M_{1}=\left[T^{3}/3,T^{2}/2;T^{2}/2,T\right]$
Measurement noise intensities	$\sigma_{r}=10\;m,\sigma_{\theta }=3.1\;mrad$
Initial measurement noise covariance matrix	$R_{k}=diag\left(\left[\sigma_{r}^{2},\sigma_{\theta }^{2}\right]\right)$
Initial state of the maneuvering target	$X_{0}={\left[1000\;m,200\;m/s,8000\;m,10\;m/s,5{}^{\circ}/s\right]}^{T}$
Initial state covariance matrix	$P_{0}=diag\left(\left[100\;m^{2},10\;m^{2}/s^{2},100\;m^{2},10\;m^{2}/s^{2},0.001\;rad^{2}/s^{2}\right]\right)$

## Data Availability

Not applicable.
